# Spatial hand representation in anorexia nervosa: a controlled pilot study

**DOI:** 10.1038/s41598-021-99101-6

**Published:** 2021-10-05

**Authors:** J. Verbe, P. G. Lindberg, P. Gorwood, L. Dupin, P. Duriez

**Affiliations:** 1 CHRU Tours, Clinique Psychiatrique Universitaire, Tours, France; 2 Institute of Psychiatry and Neurosciences of Paris, Unité Mixte de Recherche en Santé (UMRS) 1266 Institut National de la Santé et de la Recherche Médicale (INSERM), Université de Paris, 102-108 rue de la Santé, 75014 Paris, France; 3 GHU Paris Psychiatry and Neuroscience, Clinique des Maladies Mentales et de l’Encéphale (CMME), Sainte-Anne Hospital, 75014 Paris, France

**Keywords:** Neuroscience, Physiology, Diseases, Medical research, Neurology, Signs and symptoms

## Abstract

Body representation distortion (BRD) is a core criterion of Anorexia Nervosa (AN), and is usually assessed subjectively, focusing on body shape. We aimed to develop a new assessment to evaluate body representation independently from socially-mediated body image, on a body part with low emotional salience (hands). In a monocentric open label pilot study, we measured hand representations based on explicit (verbal) and implicit (tactile) instructions. Participants, with eyes closed, had to point targeted locations (knuckles and nails of each finger) based on verbal instructions and tactile stimulations to evaluate body representations respectively. Ratios between hand width and finger length were compared between AN (n = 31) and controls (n = 31) and correlated with current body mass index, AN subtype and disease duration. To control that hand distortion was specific to body representation, we also assessed object representation. Hand representation’s width/length ratio was significantly increased in patients with AN, whereas no difference was found in object representation. We found no correlation between hand wideness and clinical traits related to eating disorders. Our results propose that BRD is not limited to body parts with high emotional salience, strengthening the hypothesis that anorexia nervosa is associated with profound unspecific BRD.

## Introduction

Anorexia nervosa (AN), a concerning eating disorder (ED) in many countries, still lacks understanding of its physiopathology. Women are more affected by this disease with a sex ratio of 1/9, and the mortality rate is the highest among psychiatric disorders^1,2^. AN is characterized by a number of clinical symptoms, including body representation distortion (BRD), sometimes called “dysmorphophobia”^3^. How we represent our body has consequences for definition of the self and mental health. In AN, body dissatisfaction and weight concern are linked to body representation, and take part in the development and the maintaining of the disease^4^. BRD often persists after weight restoration^5,6^ and taking it into account in care for AN remains a clinical challenge^7^. In AN, body distortion usually occurs through an overestimation of general body size^8,9^.

Interestingly, body representation (tested using verbal instructions to locate targets on the hand hidden from view) is also distorted among healthy participants^10^. The resulting shape of hand representation shows an overestimation of hand width and an overall underestimation of finger length^10^. Similar distortion has been found^11^ for implicit body representation based on tactile instructions: participants had to indicate the location of different tactile stimuli applied on the hand on a silhouette of their hand. Moreover, these biases appeared to mirror known characteristics of primary somatosensory cortical maps^11^.

Different experimental paradigms have been used to investigate various aspects of body representation such as the rubber hand illusion^12^, localization task based on tactile or verbal instructions as described above^10,11,13^ or indirect behavioural measures^14^.

In patients with AN, body image concern has been widely studied^4,15^ and body image distortion is generally accepted as a main diagnostic criterion, but the origin of body image alteration has not been identified yet. BRD could be due to multisensorial integration impairments of body representation. Several studies support the idea that AN is associated with deficits of somatosensory integration^14,16–22^: tactile perception, vision, haptic perception and even action-oriented tasks seem to be impaired in AN. Indeed, perturbations in multisensory integration regarding the body itself may be an endophenotype of AN, and patients with this disease may use different strategies from healthy individuals in evaluating their own weight and size (for a review, see Gaudio et al.^23^). Excessive or lack of focus on exteroceptive input (i.e., sensory signals from outside, such as vision) may play a role in AN patients’ body representation distortion. The allocentric-lock hypothesis posits that patients with AN have a defective multisensory integration and consequently have difficulties to update body representation after weight loss^24^. Consequently, incoming perceptual or sensory signals differ from the stored information, which produces a mismatch that could contribute to pathologically exaggerated body representation distortions.

Patients with AN usually describe body concern focused on hips, thighs, abdomen (feeling too fat and seeing themselves too large). Some approaches to assess BRD evaluate representation of these body parts, asking patients to place on a wall indicators reflecting the width of their waist and hips, the latter being compared with actual sizes^25^. Questionnaires used in ED, such as the BSQ (body shape questionnaire)^26^ and the EDI (eating disorder inventory)^27^, also focus on these body parts. However, as body representation disorders could be caused not only by cultural pressure and current thinness ideal, but also by a multisensorial integration defect of body representation, we hypothesized that BRD is not limited to body parts with high emotional salience such as hips, thighs or abdomen, and that it could be interesting to assess BRD in other body parts, such as hands. Indeed, hands are commonly assumed to be “less emotionally salient” in ED^22^, and patients do not usually report being disturbed by their hands’ appearance.

Nowadays, body representation disorder is subjectively measured, with feminine shapes (shape’s silhouette), anthropometric diameters (rope test), or with numerical tools such as virtual reality^28^. While these approaches focus on conscious body image, in the present study we use a more objective approach which measures implicit distortion. The aim was to focus on hand representation based on explicit and implicit instructions in motionless AN patients, hypothesizing that patients with AN have a wider representation of their hand compared to healthy controls. We used a method inspired from localization tasks described above^10,11^: participants, with eyes closed, had to point on a tablet placed above their hand the location of their hand’s knuckles and nails following verbal (explicit) or tactile (implicit) instructions.

## Methods

In a monocentric open label pilot study, patients were recruited in a specialized eating disorder clinical unit. Healthy controls were recruited in the Clinical Research Centre of the same hospital.

### Participants and clinical assessment

Included participants were older than 18. Participants with hand injuries, pathologies affecting tactile sensitivity (as diabetes) or neurological or cognitive disorders were excluded from the study. AN patients were assessed by a psychiatrist following DSM5 criteria. Healthy controls (HC) were recruited among volunteers taking part in another validation trial of the task. Exclusion criteria for HC were: self-declared psychiatric diagnosis, neurological or cognitive disorder with sensorimotor impairment, assessed by an interview with the participant.

The clinical data collected for AN participants were: current BMI, minimal BMI, subtype of AN, disease duration.

Participants were matched for age, as hand width representation reduces with age (Dupin et al., submitted for publication). They were not matched for sex as there is no known differences in hand’s representation between male and female (Dupin et al., submitted for publication). AN patients were assessed at the beginning of outpatient treatment or during inpatient treatment.

The number of participants was established from a power calculation (power = 0.8, alpha = 0.05) on preliminary sample of 20 participants by group to identify a potential difference between groups for tactile (HC mean: 1.26 (S.D. 0.36), AN mean: 1.53 (S.D. 0.52, n = 29) and verbal (HC mean: 1.34 (S.D. 0.48), AN mean: 1.90 (S.D. 0.63), n = 11) conditions. Some hospitalized patients (N = 5) had an initial diagnosis of AN but gained weight during the course of treatment, resulting in normal weight at the time of the testing. Other symptoms of AN were still present. These patients did not differ on any of the outcome variables of the current experiment, and we chose to include them in the AN group.

### Experimental design, procedure and conditions

The study procedures were verbally explained at the beginning of the session. The evaluation lasted approximately 30 min. The different conditions (implicit with tactile command, explicit with verbal instruction, and object) were assessed on the non-dominant hand, so the participant pointed with their dominant hand. The order of the conditions was randomized between participants.

**Figure 1:**
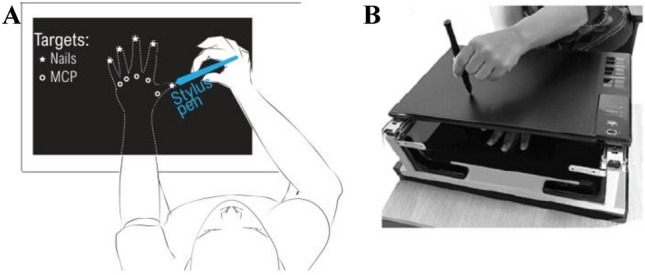
(**A**) Schematic top-view of the set up used for assessing body representation. The left hand of the participant is positioned under the graphic tablet while they pointed with eyes closed to a target—nail or metacarpophalangeal joint (MCP)—indicated by the experimenter using the stylus pen (without somatosensory or visual feedback). (**B**) Picture of the device.

Participants were seated on a chair. A graphic tablet was placed above the participant’s non-dominant tested hand (approximately 6 cm; Fig. 1A,B). The tested hand was positioned palm down in the sagittal axis of the shoulder. Participants were instructed not to move their tested hand during the experiment. They had to keep their eyes closed during the different tasks, since non-informative vision can affect tactile localization^29–31^ and also influence participant responses by using visual reference and/or explicit knowledge of hand characteristics^32^.

At the beginning and the end of the experiment, the physical position of the target points of the hand were recorded (PHYSICAL). The task was to point with the stylus pen on the graphic tablet directly above where they perceived the target indicated by the experimenter, without time limitation.

There were 10 possible targets, randomized and repeated 3 times per condition: the five nails and the five metacarpophalangeal joints. Then the coordinate was recorded from the graphic tablet.

The experimenter could indicate the target verbally (VERBAL condition) or use tactile stimulation of the target using a stick with a foam tip (TACTILE condition).

**Figure 2:**
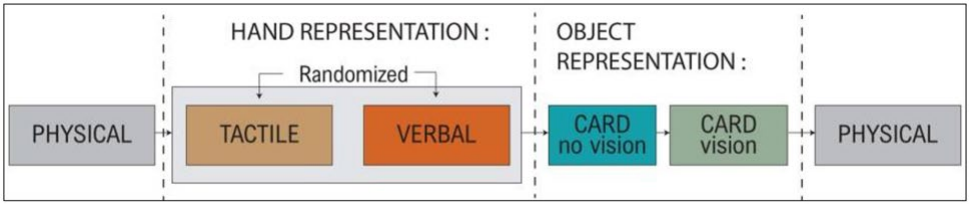
Design of the experiment: 3 conditions concerning the measure of the hand representation (PHYSICAL, TACTILE, VERBAL) and 2 concerning the representation of an object in external space as control (CARD-no vision, CARD-vision).

After VERBAL and TACTILE conditions, object distortion was assessed (CARD condition). This CARD condition was used to control that the potential body distortion was specific to the body and not due to a general pattern of space distortion with eyes closed. In this condition, participants had to successively point the four corners of an imagined asked card whose size is standard and known by the participant (credit card format, 8.56 × 5.4 cm, each corner repeated 3 times). The task was done twice: first with eyes closed (CARD no vision) and then with eyes open (CARD vision). The distortion was the difference between the card with eyes open and eyes closed (see Fig. 2. for a summary of the conditions).

### Data analysis

The main measure for each participant was the ratio between normalized hand width and finger length (noted rWideness for relative—i.e. normalized—wideness of the hand). For each condition (VERBAL, TACTILE, PHYSICAL) and each participant, the barycentre of each target (nail/metacarpophalangeal joint of each finger) repetition was computed. We used this barycentre as the responded location of the target.

#### Normalized hand width

Hand width was computed as the distance between metacarpophalangeal joint of the little finger and the thumb. Hand width in TACTILE and VERBAL conditions was divided by PHYSICAL hand width in order to normalize data between participants. The length of each finger was computed as the distance between the nail and the metacarpophalangeal joint of each finger. Then the mean of the five finger lengths was computed.

#### Normalized finger length

Finger length in TACTILE and VERBAL was divided by PHYSICAL finger length in order to normalize data between participants.

The relative wideness of the hand corresponds to the ratio between hand width and finger length of the representation both normalized over the corresponding physical hand extent. In other words, rWideness is the relative wideness of the representation compared to the one of the real hand. rWideness was computed so that a value of 1 means that the representation has the same ratio between hand width and length of the participant’s physical hand. A rWideness lower than one means that the representation of the hand is narrower than the physical hand and a ratio greater than 1 means that the representation is wider.

#### Normalized CARD width and length

CARD width was the mean distance between the two edges in the frontal plane while CARD length was the mean distance between the two edges in the sagittal plane. Width and length in CARD no vision was divided with CARD vision in order to obtain the distortion of the card with eyes closed compared to what participants « think » the real size of the card is.

### Statistical analysis

Data analyses were computed using Wolfram Mathematica 11.3 for distance computation and R for statistical analyses.

We used Shapiro–Wilk test to test for data normality. We found that rWideness did not follow normal distribution for VERBAL and TACTILE conditions. Consequently, we used Wilcoxon signed rank test to analyse between-group differences, corresponding effect size r_e_ were computed for significant results as r_e_ = Z/(N)^1/2^. For the AN group, we performed non-parametric correlation analyses (Spearman correlation) between rWideness and clinical data current BMI, minimal BMI, duration of the ED, and subtype of AN (binge/purge or restrictive). We did not record BMI for HC group.

### Apparatus

The device consisted of a graphic table (HUON WH1409) connected to a PC through USB. The graphic tablet (Fig. 1B) was mounted on a support by the mean of two slides so that the tablet could be slid away from the hand in order to locate the physical position of the fingers. The software used to record data was developed in C++.

### Ethic statement

All study procedures were in accordance to the Declaration of Helsinki and approved by the French ethics committee “Comité de Protection des Personnes” (2017-A01875-48). Each participant received an information letter about the study. In accordance with the Helsinki declaration, written informed consent was obtained from each participant before inclusion.

## Results

### Population

We tested 62 participants, 31 female patients with AN (n = 14 binge/purge type, n = 17 restrictive type) and 31 HC whom we matched as closely as possible for age and handedness (3 left-handed in the AN group, 2 in the HC group). Mean body mass index (BMI) in the AN group was 15.49 ± 2.3 (mean ± SD) kg/m^2^ (range 12.4–20.6), and mean lower BMI was 13.44 ± 1.42 (mean ± SD) kg/m^2^ (range 9.9–16).

Mean age of AN patients was 27.19 ± 8.99 (mean ± SD) years (range 18–51) and mean ED duration was 9.10 ± 8.47 (mean ± SD) years (range 1–37). Mean age of healthy controls was 27.74 (± 8.2) years (range 18–47). Table 1 lists demographic and clinical characteristics of the sample. HC and AN did not differ regarding age (*p* = 0.1863). There were 5 males in the HC group, none in the AN group.

**Table 1:** Demographic and clinical characteristics.

	AN (n = 31)			HC (n = 31)			p-value
	M	SD	Range	M	SD	Range	
Age (years)	27.19	8.99	18–51	27.74	8.20	18–47	0.1863
Laterality	3 LH, 28 RH			2 LH, 29 RH			
BMI (kg/m^2^)	15.49	2.30	12.4–20.6	–	–	–	–
ED duration (years)	9.10	8.47	1–37	–	–	–	–
Minimal BMI	13.44	1.42	9.9–16	–	–	–	–

LH: left-handed, RH: right-handed, AN-BP: anorexia nervosa binge purge subtype, AN-R: anorexia nervosa restrictive subtype.

### Ratio of the body spatial representation tasks

The relative wideness (rWideness, see “Methods”) of the hand corresponds to the hand width/length ratio of the representation compared to the width/length ratio of the physical hand, that is the relative wideness of the representation compared to the one of the physical hand.

As previous studies found shorter finger and wider hand representation in healthy populations, we controlled if rWideness was greater than 1. We found that rWideness was significantly greater than 1 in both conditions in the HC group (Tactile: median = 1.23, Z = − 4.19, *p* < 0.001; r_e_ = 0.53 Verbal: median = 1.28, Z = − 3.39, *p* < 0.001, r_e_ = 0.49, one sample Wilcoxon signed-rank tests) and in the AN group (Tactile: median = 1.44, Z = − 5.24, *p* < 0.001, r_e_ = 0.67; Verbal: median = 1.77, Z = − 6.12, *p* < 0.001, r_e_ = 0.77, one sample Wilcoxon signed-rank tests), coherent with the general pattern of distortions previously found in healthy populations.

**Figure 3:**
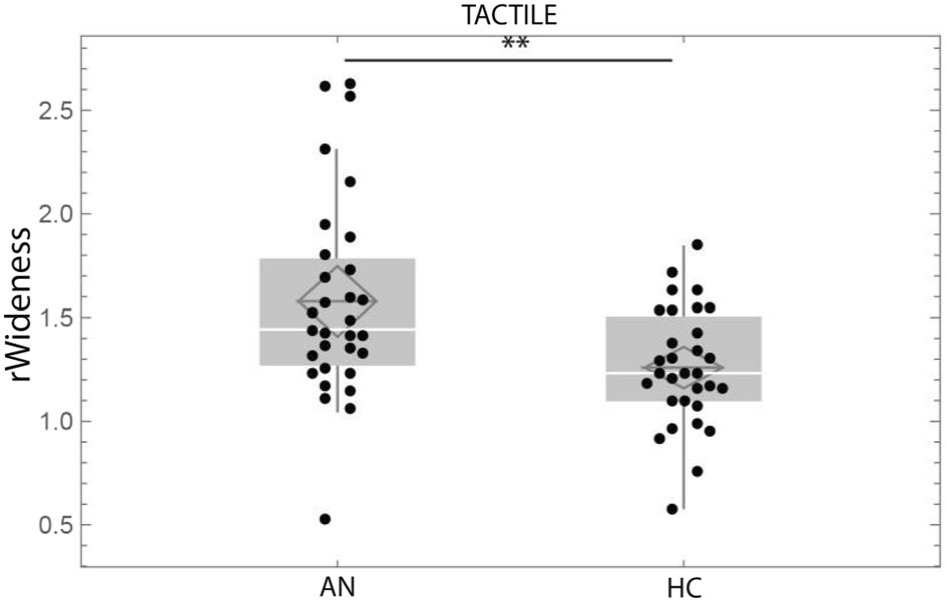
Boxplot of rWideness in the Tactile condition, in AN vs HC. rWideness Tactile: ratio hand width on hand length in the Tactile condition. **p < 0.01, ⊇: mean, white line: median, grey box displays the interquartile range, whiskers indicate variability outside the upper and lower quartiles.

**Figure 4:**
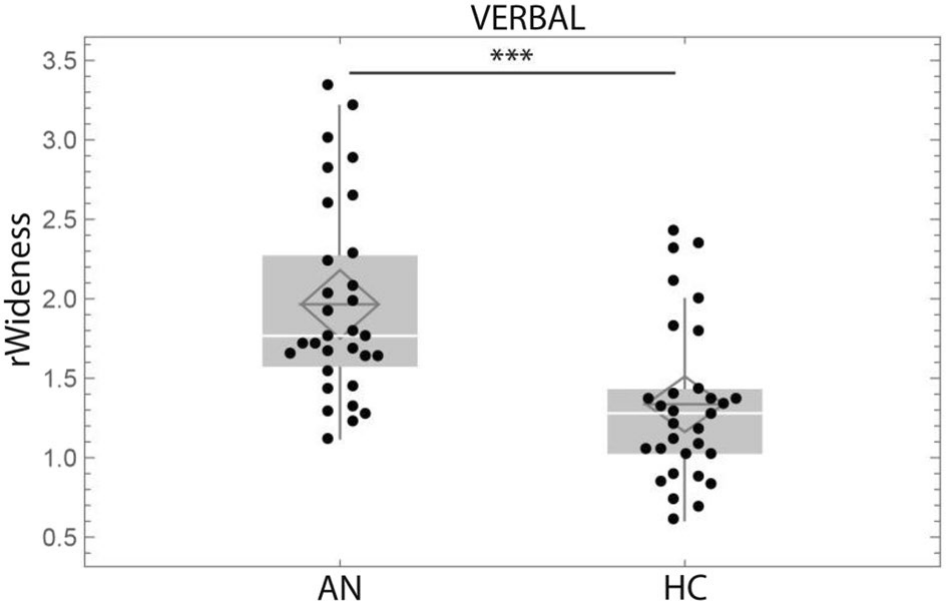
Boxplot of rWideness in the Verbal condition for AN and HC. rWideness Verbal: ratio hand width on hand length in the Verbal condition. ***p < 0.001, ⊇: mean, white line: median, grey box displays the interquartile range, whiskers indicate variability outside the upper and lower quartiles.

We compared rWideness between AN and HC groups (Table 2) and found significantly greater rWideness for AN compared to HC in both TACTILE (Z = − 2.98 *p* = 0.003, d′ = 0.5956, r_e_ = 0.38, Wilcoxon signed-rank test, Fig. 3) and VERBAL conditions (Z = − 3.58, *p* < 0.001, d′ = 0.7158, r_e_ = 0.45, Wilcoxon signed-rank test, Fig. 4). We found no significant difference between AN and HC for the control task CARD (object distortion) (Z = − 0.30, *p* = 0.765, Wilcoxon signed-rank test). These results indicate that the increased distortion in AN is specific to the body since it does not affect object representation.

**Table 2:** Tactile and verbal ratio for AN and HC. Wilcoxon signed-rank test between AN and HC.

	Tactile			Verbal			CARD		
	Median	SD	IQ1-IQ3	Median	SD	IQ1-IQ3	Median	SD	IQ1-IQ3
AN	1.4433	0.4816	1.2885–1.7671	1.767	0.6120	1.595–2.264	0.9132	0.2518	0.8260–0.9848
AN-BP	1.5471	0.4993	1.2593–1.7238	1.7457	0.7056	1.6489–2.1823	0.8691	0.1389	0.8171–0.9681
AN-R	1.4178	0.4769	1.3181–1.8011	1.7996	0.5445	1.5505–2.2818	1.0069	0.2913	0.8494–0.9972
HC	1.2325	0.2839	1.0984–1.4771	1.2797	0.4960	1.0274–1.4228	0.9662	0.1695	0.8449–1.0400
p	0.0029			0.0003			0.7646

There was a significant positive correlation between the rWideness Tactile and Verbal in the Healthy Control group (Spearman ρ = 0.50, *p* = 0.005) but not in the AN group (Spearman ρ = 0.24, *p* = 0.187). The rWideness Tactile and Verbal significantly differed in the AN group (Z = − 3.16, *p* = 0.002, r_e_ = 0.40, Wilcoxon signed-rank test) but not in the HC group (Z = − 0.32, *p* = 0.750, Wilcoxon signed-rank test).

The difference between Verbal and Tactile condition was significantly greater in the AN group (median = 0.34) compared to HC (median = − 0.02, Z = − 2.21, *p* = 0.027, r_e_ = 0.28, Wilcoxon signed-rank test).

Analysis showed no significant differences between AN-BP subgroup (n = 14) and AN-R subgroup (n = 17) for Tactile (Z = − 0.3326, *p* = 0.7395, Wilcoxon signed-rank test) and Verbal conditions (Z = − 0.02, *p* = 0.984, Wilcoxon signed-rank test). There was also no difference between early weight-recovered AN patients (N = 5) and other AN patients (N = 26) for Tactile (Z = − 0.65, *p* = 0.514, Wilcoxon signed-rank test) and Verbal conditions (Z = − 1.30, *p* = 0.195, Wilcoxon signed-rank test).

### Correlation of behavioural measures and clinical data

We found no significant correlation between rWideness in the Tactile condition and current BMI (Spearman ρ = 0.07, *p* = 0.724), minimal BMI (Spearman ρ = − 0.09, *p* = 0.6246), or ED duration (Spearman ρ = 0.01, *p* = 0.950) in the AN group. Similarly, we found no significant correlation between rWideness Verbal and current BMI (Spearman ρ = − 0.18, *p* = 0.323), minimal BMI (Spearman ρ = − 0.27, *p* = 0.139), or ED duration (Spearman ρ = 0.17, *p* = 0.348).

## Discussion

In this pilot study, we used a novel method and device to evaluate body representation distortion in a population of patients with anorexia nervosa and compared it to that in healthy controls. We aimed to develop a new tool to evaluate body representation, the most independently from socially-mediated body image as possible, in order to investigate more specifically the physiopathology of body representation distortion within eating disorders.

We found wider hand representation than the physical hand in both groups and conditions. However, hand relative wideness of patients with AN was significantly increased compared to controls, in both the verbal (explicit) and the tactile (implicit) conditions. There was less increased hand relative wideness in the Tactile condition compared to the Verbal condition in the AN group. Another difference between groups was that tactile and verbal hand distortions were correlated in controls, but not in the AN group. In contrast, no difference between groups was found for object representation. Finally, there was no correlation between hand representation relative wideness and BMI or ED duration for AN patients in the study. AN patients with recent normal weight showed the same distortion as underweight patients.

Hand representation is not identical to the physical hand, even in healthy persons. Previous studies on hand size estimation found that hand representation based on verbal instruction (explicit) was wider than the physical hand in healthy persons, but they did not report the ratio between hand width and finger length^10,11^. We also found that body representations based on explicit and implicit instructions were distorted in healthy participants, with an increased hand relative wideness in this group. Both conscious body image and sensory perception correlates differ from the physical hand; however, in AN patients, this distortion is stronger.

An overestimation of hand width using a caliper with pointers was also previously found in AN patients^12^. Only the explicit hand width representation was evaluated (corresponding to our verbal condition, but we measured the relative wideness), and they did not compare the estimated hand width of AN patients with that in healthy controls. This experiment used the rubber hand illusion with therapeutic purpose: it was found that the rubber hand illusion improved estimation of hand width in AN patients, the hand width being less overestimated after the rubber hand illusion.

The overestimation of hand wideness was less substantial in the Tactile condition compared to the Verbal condition: in other words, conscious hand representation is more impaired than pure implicit (or somatoperceptive) hand representation in the AN group. This difference between the two representations in AN can be related to the fact that a lack of somatoperceptive feedback increases the distortion. Clinically, patients often use body checking behaviours, such as trying to feel their bones on specific parts of their body, pinching or wrapping a hand around the stomach or thigh, etc. These behaviours could be related to attempts to balance their impaired explicitly-instructed body representation in the verbal condition. Moreover, both representation distortions based on implicit and explicit instructions correlated in the HC group and consequently, in this group, both representations seem to rely on similar processing and share neural mechanisms. In the AN group, this was not the case: implicitly and explicitly-instructed body representation distortions were not correlated, indicating an imbalance between the two body representations.

Both explicitly and implicitly instructed representations were unrelated to BMI. Undernutrition is linked to several AN symptoms, but it does not appear to be related with BRD in our study. Our results are congruent with the clinical observation that BRD often persists after weight restoration, and with the allocentric-lock hypothesis (i.e. related to difficulties to update body representation after weight change). Patients may have difficulties in updating their body representation along with the weight changes, due to their defective multisensory integration. BRD is a strong risk factor of relapse and may play a main role in remission^4,7^.

In this study, both explicitly and implicitly-instructed representations were unrelated to ED duration. The patient sample size was limited, and only composed of adults, with a wide ED duration range (1–37 years). Further research with more participants and younger patients is needed to investigate whether similar distortions are present early in AN and to confirm independency with ED duration. An endophenotype of AN, shared by patients and unaffected relatives, is supported by recent work; it usually concerns the intense and compulsive exercise exhibited by most patients and neurocognitive features^33–35^. Body representation distortion could also take part in it. Further research using the hand representation spatial task in recovered AN and relatives is necessary to precise whether this body representation distortion is a stable vulnerability trait for eating disorders or if it is a symptom occurring during the period of the illness and lingering after weight restoration before improving.

Our results add to the growing evidence that somatoperception and conscious body image are impaired in AN, and strengthen the hypothesis that AN has profound and unspecific body distortion. The method used in this study can objectively evaluate these impairments with a simple test which is easy to perform, replicable and potentially compatible with neuroimaging. The assessment used in this study provided an indirect/unconscious measure of body representation as hand is not one of the most commonly affected body parts reported by patients affected by AN. Nevertheless, the assessment of the hand showed strong distortion, unveiling a new approach to measure body representation distortion in AN.

### Limitations

First, our sample was not homogeneous in term of weight status, and some patients had recently gained weight. In our sample, we did not find any evidence for an effect of weight recovery, however, assessing hand representation in a third group made of weight-recovered AN patients would be necessary to confirm this observation. Second, participants did not have a subjective evaluation of body representation (with questionnaires). Third, some of our control subjects were men, while there were only women in the AN group. However, no gender difference in spatial hand representation distortions was found in previous work (Dupin et al., submitted for publication). Therefore, there should be no major impact of this difference between groups on the results. The originality of the assessment is also a limitation. Similar measurements on other parts of the body could have better characterized the specifics of these implicit hand measurements and should be incorporated into future studies.

## Conclusion

Using a novel method to evaluate body representation in AN, we targeted a low emotionally salient body part (i.e. hand) in AN patients and controls to assess body representation using explicit and implicit instructions. We found that hand representation was wider in patients with AN in both representations than age-matched healthy controls. Our results support a body representation disorder hypothesis and show that BRD is not limited to body parts with high emotional salience, as it also concerns hands. Our method is easy to use in clinical practice, allows for immediate quantification and implicit assessment. It allows quantification of body representation in both clinical care and research concerning anorexia nervosa and eating disorders.

## Data Availability

Data used in this work are available upon request to the corresponding author.
